# Thyroid isthmus agenesis associated with solitary nodule: A case report

**DOI:** 10.1186/1757-1626-1-211

**Published:** 2008-10-03

**Authors:** Alberto Schanaider, Paschoal Josias de Oliveira

**Affiliations:** 1 Experimental Surgical Center, CCS, Bloco J, 2nd floor, Universitary City, UFRJ, Ilha do Fundão, Rio de Janeiro, Postal code/CEP: 21944-970, Brazil

## Abstract

**Background:**

Agenesis of the isthmus associated with nontoxic solitary nodular goiter is a rare congenital anomaly. Imaging data exceptionally has been previously reported in the English language literature.

**Case presentation:**

Preoperative assessment of a 22-year-old white man patient showed an asymptomatic nodule of the thyroid at the left lobe, measuring 2.3 × 1.5 cm in diameter without regional lymphadenopathy. At surgical exploration it was seen an absence of the thyroid isthmus.

**Conclusion:**

Thyroid isthmus agenesis does not cause clinical symptoms by itself and most of the times the diagnosis is incidental due to the existence of other thyroid pathology.

## Background

An isolated thyroid isthmus agenesis is very uncommon. The true incidence of this condition is very difficult to be determined, since the patients have been found in a euthyroid state. Thyroid isthmus agenesis does not cause clinical symptoms by itself and most of the times the diagnosis is incidental due to the existence of other thyroid pathology [1].

## Case presentation

**Figure 1:**
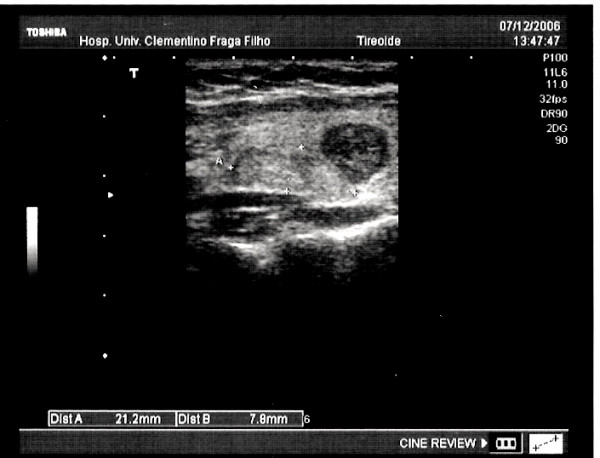
Ultrasonography of the thyroid gland with visualization of heterogenous nodule in the left lobe.

**Figure 2:**
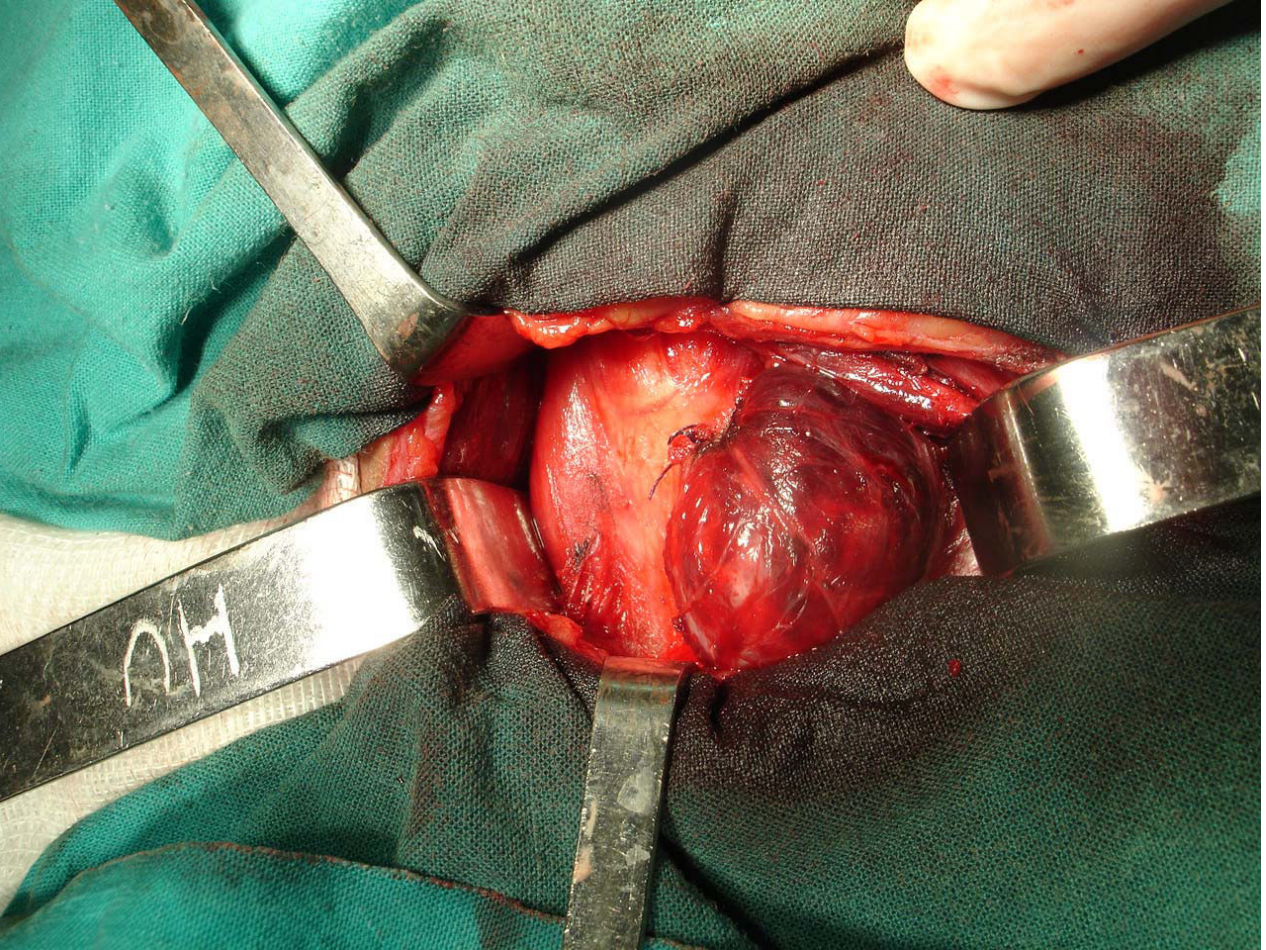
**After neck surgical exploration it was seen both lobes and absence of the thyroid isthmus.** The left lobe with a solitary nodule was exposed after the superior pedicle ligature.

In 2005 a 22-year-old white man patient was referred for treatment of arterial hypertension. On his first physical examination it was seen an asymptomatic nodule at the medium part of the left lobe, measuring 1.5 cm in diameter without regional lymphadenopathy. The ultrasound exam showed a topical thyroid with a solitary 2.3 × 1.5 cm heterogenous nodule with areas of cystic degeneration. The echo/doppler investigation displayed a peripheral flux. On this occasion a fine-needle aspiration cytology was made and results showed papilliferous cells without malignancy features. Immunohistochemical study for cytokeratin-19 marker was positive. One year later, a second ultrasonography showed the enlargement of the nodule by reaching 2.7 × 2.5 cm (Figure 1). Thyroid isthmus agenesis was found (Figure 2) during the access to the gland and a left lobectomy was performed. Histologic examination yielded to the diagnosis of nodular goiter.

## Discussion

Most of the descriptions in the literature about congenital thyroid abnormalities are related with hemiagenesis which include one lobe and sometimes the isthmus. Thus congenital thyroid anomalies can involve one or both lobes and the isthmus may be present or not. Over 50.000 thyroid patients operations at Mayo Clinic during 40 years, only five cases of hemiagenesis were described [1]. Mirkosch et al [2] during a 9 years studied 71 500 patients who underwent thyroid investigation. Ten of them had isthmus hemiagenesis. Others surgical studies reported an incidence nearby one in 2000 [1,3]. Melnick [1] and Mikosch [2] on two reviews of available world literature found, respectively a total of 94 and 256 cases of thyroid hemiagenesis but among these cases the isthmus was absent in only 50% of the patients where the isthmus was specifically mentioned.

There are a variety of diseases associated with thyroid partial agenesis and most of them are cases of hyperthyroidism or hypothyroidism in iodine deficient area and also nontoxic nodular goiter [1,2,4,5].

Mutation of genes has been involved in thyroid morphogenesis [5] but failure of the isthmus fusion in the midline may be the principal cause of an isolate isthmus agenesis.

Ultrasonography is still the key investigation to diagnose isthmus agenesis but the presence of other pathological conditions diverts the attention and misleads the diagnosis. The use of both computed tomography (CT) and magnetic resonance imaging does not offer best results than ultrasonography itself which can be performed easily and quickly, with less cost and no radiation risk as seen at CT. Furthermore taking into account the performance of these three diagnostic methods to identify the morphology of thyroid, the results are equivalent.

In asymptomatic patients with nodular goiters fine-needle aspiration biopsies and eventually immunohistochemistry tests are useful to support the medical decision but when agenesis is present the importance of pre-operative differentiation between benign and malign lesion is critical, considering the surgical procedure and the possibility of impairment of the thyroid function.

## Conclusion

Isolate agenesis of the isthmus associated with nontoxic solitary nodular goiter seems to be an unusual condition and imaging data exceptionally has been previously reported in the English language literature.

## Competing interests

The authors declare that they have no competing interests.

## Authors' contributions

AS performed the surgery and PJOJ made substantial contributions to acquisition of data. Both had important contribution in writing the manuscript. All authors read and approved the final manuscript.

## Consent

Written informed consent was obtained from the patient for publication of this case report and accompanying images. If necessary a copy of the written consent is available for review by the Editor-in-Chief of this journal.
